# The Making of a Surgeon

**Published:** 1905-11

**Authors:** George Ryerson Fowler

**Affiliations:** Brooklyn, N.Y., Professor of Surgery, New York Polyclinic (Emeritus)


### The Making of a Surgeon.

BY GEORGE RYERSON FOWLER, M. D., Brooklyn, N. Y.
Professor of Surgery, New York Polyclinic (Emeritus)

[*Brooklyn Medical Journal*]

WHEN a master seeks an apprentice, if he be in love with his work and earnestly seeks to initiate him who is to follow in his footsteps, he will select one whose intelligence, as well as knowledge already gained, fits him for the calling in hand. When such a selection is made, the master sets himself about the task of making a craftsman of the material furnished him.

He does not commence by delivering himself of a flow of words, at certain stated intervals, that have little meaning to his apprentice. He knows full well that his own and the apprentice's time will be wasted, and that every hour thus spent will not only be profitless, but will tend to detract from the interest that his apprentice takes in the work. Even though the master be an accomplished lecturer, and deliver himself with the aid of the simplest notes, yet will the labor be in vain. If he seeks to make a craftsman, he will keep before the apprentice the best examples of the results of his handicraft, and by precept and example seek to embue his receptive mind with the different steps of the processes whereby the final and complete machine is produced, and guide his hand through the simple and finally to the intricate stages of the workmanship.

If, as in the modern methods of making skilful workmen, the apprentice enters the temple of his chosen calling through the industrial school and a course in mechanical engineering, he will have learned the physical properties of metals, their behavior under varying conditions of heat and cold and the theories of tensile and cross-strain; of environment that hastens deterioration as well as the evidences of the presence of the latter, and the means of ensuring prolonged service. All of these he will have learned in the recitation room and in the laboratory, supplemented, as time goes on, by practical demonstrations at the forge and at the bench and vice.

But all the knowledge gained of the qualities of metals, from the rough chemistry of the mines to the delicate manipulation of the crucible in the assaying room, will not make a machinest of the apprentice any more than will a knowledge of how best to cultivate trees make a cabinet maker. While with this knowledge combined with the acquirement of the manipulative skill to fashion a locomotive or a watch a master workman is produced, the one without the other produces in the one case a theorist without practical application when the actual work is to be done, and in the other case a mere human machine.

With his store of theoretical knowledge the apprentice must first fetch and carry. He must clean the rough castings, mingle with the more advanced apprentices, and now and again adjust a bolt and tighten a nut. He is taught how to use his hands and brain in combination, and with his fundamental knowledge of the principles of the craft, and learning by actual doing under the eye of the master mechanic or of that of an advanced craftsman, he passes on from the simplest to the most complicated tasks of the art. From this time on his course is one of constant progress toward a higher plain of efficiency, until, with the development and exhibition of added qualifications of loyalty and resources he becomes the trusted director of the master's work. Finally, with ripened mind and trained hand he develops into an original thinker, a chief craftsman, a leader of his fellows.

Turn we now to the making of a surgeon. Time was when the professor of surgery, entering the lecture room, bowed formally to the assembled students with scarcely a familiar face confronting him. He proceeded to drag his audience after him as he waded through the hour's discourse, either reading from the copied pages of some so-called authority, or reciting from memory reinforced by the well-worn notes of previous and similar didactic efforts. Here and there a more industriously inclined student busied himself taking notes of what he conceived to be the important points of the subject. Others attempted by a fixed stare to prop up the unwilling eyelids, while others again yielded to the seductive wooings of Morpheus until awakened by the formal applause that marked the end of the hour, and that was intended less for appreciation of the lecturer's efforts than for relief of the hum-drum environment. Add to this knowledge on the part of the more knowing ones that the professor's grasp of the subject lectured upon, which, for instance, is that of the treatment of wounds, is less than his knowledge of the puerperal dose of ergot, and the farcical character of the whole performance is glaringly apparent.

The only relief of this tedium was the clinical lecture, delivered perhaps twice a week, in the hospital ampitheatre. The latter was crowded from floor to dome with students in every stage of development, from first-year pupils to graybeards taking a post-graduate course, these watching a spectacular performance in which an anesthetised patient and a flourish of instruments figured prominently. Those in the front row only knew what was happening, or heard what the professor said. As to the history of the patient and his subsequent fate only the hospital records could speak, and these gave forth no hint.

And so the average medical mill ground out its yearly grist of surgeons! What with the spirit of commercialism and the curse of cliqueism to sap its vitality, and the absence of community of interest of the professional staff to bar the march of progress, it was reserved for the didactic lecture and the spectacular clinic to hold the medical college up to the ridicule of the educational world. From its walls the medical student stepped forth with a rudimentary and superficial and wholly theoretical knowledge of the science and art of surgery. Perhaps, after years spent in general practice, and if his line were cast in places where it was necessary for him to cultivate surgical skill, it might happen that he would develop the essential qualifications of a successful surgeon. In this, however, he would be handicapped by the absence of the guiding hand of a master, although the advantages of a previous large experience in general practice to the surgical practitioner are not to be underestimated in this connection.

The student of surgery should be one whose tastes, inclinations and interest lead him in the direction of this special field of work. In selecting this branch of the profession he should remember, however, that the internist is in much greater demand than the surgeon. If he is attracted by the glare of the footlights and the brilliancy of the stage-setting that seems in the mind of the average student to go along with a successful surgeon's career, let him have a care, lest the tragedy is far too real to his liking; that the tinsel of the stage is not exchanged for the sombre drapings of woe; that the smiling audience from whom he expected plaudits of praise is not changed to a group of grief-stricken mourners; that each expiring footlight does not mark the needless sacrifice of a human life; and, finally, that he does not feel the remorse of that other Judas who betrayed his trust for greed of gain.

Teachers of surgery are born, not made. The qualifications needed to make a successful instructor usually develop early in the life of the one who is to become a leader and teacher. These qualifications are more easily recognised when present than described. They consist in the main of an ability to recognise and grasp the important points of a subject, to arrange these systematically and in the order of their value, and to present them in an intelligible and attractive manner.

As to the methods of imparting instruction, class-room conferences and recitations may well replace the didactic lecture and general faculty quiz; section work at the hospital should take the place of the public clinic, and the surgical laboratory should supplement the operative work on the cadaver. These should constitute the modern methods, in addition to the curriculum of the medical course in general, for the making of a surgical apprentice.

Too much importance can not be attached to the work in what I have here termed the surgical laboratory. This department, now practically limited to experimental and original research work, should be employed as a part of the routine teaching in every medical college. Here the modern methods of applying the principles of aseptic and antiseptic surgical technic, the administration of anesthetics, trephining and exploration of the intracranial organs, operations on the eye, tracheotomy, resection of ribs, operations on the abdominal organs, particularly the technic of gastrointestinal operations, and the ligation of arteries should be taught with the lower animals as subjects. Here the provisional and definite arrest of hemorrhage may be practised, the causes and prevention of shock investigated, the phenomena of destructive and reparative processes studied, and the uses of instruments learned. Markings should be instituted based on accuracy of observation, manipulative skill, and rigid attention to details. Upon the basis of these markings a selection of those who are fitted to become surgical apprentices should be made.

Following the laboratory course as above outlined, the surgical apprenticeship of the medical student should commence. He should fetch and carry, adjust a bandage here and replace a dressing there. His workshop is the out-patient surgical department of the hospital, or the surgical clinic room of the general dispensary.

The next step of his apprenticeship is marked by his appointment to the surgical interne staff of a well-equipped hospital, and, if possible, one the work in which is exclusively, or at least largely surgical. For the cultivation of the surgical mind the atmosphere and invironment of the daily routine of work should be surgical. A mixed service is not the best for hospital internes, and this is particularly true for the surgical interne.

The different grades of service as a hospital interne mark the further progress of the apprenticeship of the surgeon. Step by step he advances, each advance being marked by new duties and increased responsibilities. From the care of cases of lesser importance he passes on to those of the most serious, and from assisting at minor operations to the performance of the latter, and thence to the operating room—all these are too well known to be dwelt on here. With this advent as the senior member and responsible head of the surgical interne staff comes the *experimentum crucis* of his surgical career. It is here that he must show that combination of prompt judgment, resourceful energy, and loyalty and devotion to duty that is the hall-mark of the true surgeon. Under the watchful eye of the master his surgical virtues and shortcomings are equally revealed and his future shaped accordingly. The first-named inspires confidence and impels his chief to entrust important emergency work to his care; the second awakens constant distrust and anxiety in the mind of the responsible head of the service.

Finally, his apprenticeship ended, he sets forth upon his life's journey. Here we must leave him. And as the *vale* springs to the lips of the master, the injunction of the great bard of Avon may well be spoken:

"This above all: To thine own self be true,
And it must follow, as the night the day,
Thou canst not then be false to any man."

